# Application of a simple quantum chemical approach to ligand fragment scoring for* Trypanosoma brucei* pteridine reductase 1 inhibition

**DOI:** 10.1007/s10822-017-0035-4

**Published:** 2017-07-07

**Authors:** Wiktoria Jedwabny, Joanna Panecka-Hofman, Edyta Dyguda-Kazimierowicz, Rebecca C. Wade, W. Andrzej Sokalski

**Affiliations:** 1 Department of Chemistry, Wrocław University of Science and Technology, Wrocław, Poland; 2 Molecular and Cellular Modeling Group, Heidelberg Institute for Theoretical Studies (HITS), Heidelberg, Germany; 3 Centre of New Technologies, University of Warsaw, Warsaw, Poland; 4 Center for Molecular Biology (ZMBH), DKFZ-ZMBH Alliance, Heidelberg University, Heidelberg, Germany; 5 Interdisciplinary Center for Scientific Computing (IWR), Heidelberg University, Heidelberg, Germany

**Keywords:** Pteridine reductase 1, PTR1, Interaction energy, Ab initio, Nonempirical model, Binding affinity prediction

## Abstract

There is a need for improved and generally applicable scoring functions for fragment-based approaches to ligand design. Here, we evaluate the performance of a computationally efficient model for inhibitory activity estimation, which is composed only of multipole electrostatic energy and dispersion energy terms that approximate long-range ab initio quantum mechanical interaction energies. We find that computed energies correlate well with inhibitory activity for a compound series with varying substituents targeting two subpockets of the binding site of *Trypanosoma brucei* pteridine reductase 1. For one subpocket, we find that the model is more predictive for inhibitory activity than the ab initio interaction energy calculated at the MP2 level. Furthermore, the model is found to outperform a commonly used empirical scoring method. Finally, we show that the results for the two subpockets can be combined, which suggests that this simple nonempirical scoring function could be applied in fragment–based drug design.

**Electronic supplementary material:**

The online version of this article (doi:10.1007/s10822-017-0035-4) contains supplementary material, which is available to authorized users.

## Introduction

Empirical computational methods are widely used in ligand discovery projects for ligand docking and computing ligand–receptor binding energetics. Docking procedures using empirical scoring functions are often found to successfully predict poses, but commonly lack sufficient accuracy to correctly rank poses or predict binding affinities [1–4]. On the other hand, rigorous ab initio quantum mechanical methods offer the possibility of more accurate calculations, but are generally too computationally costly and therefore impractical in drug design projects. Nevertheless, quantum chemical calculations may provide deeper insights into the physical nature of the corresponding interactions and lead to simpler and more robust nonempirical models. An example is the finding that for polar or charged inhibitors of phenylalanine ammonia-lyase and leucine aminopeptidase, the nonempirical first-order electrostatic interaction energy defined within perturbation theory [5] (or its multipole component [6]) alone yielded a reasonable correlation with experimental inhibitory activity data. However, such a simple model is insufficient for nonpolar receptors, like fatty acid amide hydrolase (FAAH), where inclusion of a nonempirical dispersion term, in addition to the electrostatic multipole term, was necessary to describe inhibitory activities [7]. Likewise, as noted by Lonsdale et al. [8], dispersion effects should be considered for reliable modeling of enzyme-catalyzed reactions.

While the electrostatic multipole term estimated from atomic multipole moments obtained from RHF wavefunctions scales favorably with the number of atoms *A* of the studied system as $O(A^2)$, the ab initio calculation of dispersion energy is much more computationally demanding, scaling as $O(N^5)$, where *N* is the size of the basis set and, as such, it cannot be part of a generally applicable scoring method. A computationally inexpensive empirical expression for the dispersion energy employed by classical force fields [9] might be seen as a rational substitute for the ab initio calculations [10, 11]. However, empirical dispersion appears to be associated with a non-systematic error compared to rigorous DFT-SAPT results [10]. Another drawback of the classical term seems to arise for intermonomer distances shorter than equilibrium separation, wherein empirical results deviate from the reference DFT-SAPT calculations [11]. Since such shortened intermolecular distances might result from force field inadequacy [12] or basis set superposition error [13], any method including short range intermolecular energy terms sensitive to artificial compression of intermonomer separation is inadequate for the purpose of rapid estimation of the binding energy within protein–ligand complexes.

Most attempts to derive affordable and reliable dispersion corrections have been undertaken in conjunction with density functional theory methods, which do not account for the dispersive van der Waals forces unless special corrections are added [14–16]. Pernal et al. [17] proposed an alternative approach—a dispersion function $E_{Das}$ that describes noncovalent interactions by atom–atom potentials fitted to reproduce the results of high-level SAPT (Symmetry Adapted Perturbation Theory [18]) calculations that provide state-of-the-art quantum chemical dispersion and exchange-dispersion energies. It is noteworthy that the $E_{Das}$ function demonstrated remarkable performance in describing hydrogen bonding interactions, which are governed by both electrostatic and dispersive forces [19]. The low computational cost of this approximate dispersion function and its broad applicability stemming from the lack of empirical parametrization, make the use of the $E_{Das}$ expression a promising approach to describing dispersive contributions in scoring methods suited for virtual screening. Further advantages of the $E_{Das}$ term over van der Waals 1/r$^{6}$ empirical expression discussed above are the clear physical meaning of the former and its pertinence to a wide range of intermolecular distances because of an additional higher order 1/r$^{8}$ term and an exponential damping function that is essential at short distances where penetration effects become significant.

Here, we evaluate the ability of the simple model that was previously tested for a congeneric series of inhibitors of the FAAH protein [7], to predict the activities of inhibitors targeting two different subpockets of a protein binding site, which is an important requirement for application in fragment-based drug design approaches. In this model, the ligand–receptor interaction energy is approximated by the sum of the first-order electrostatic multipole component of the interaction energy, $E_{EL,MTP}^{(10)}$, and $E_{Das}$, the aforementioned approximation of dispersion energy. The advantage of such a model is that it captures both long-range electrostatic and dispersive energy terms, while being relatively computationally efficient. The interaction energy of an inhibitor or a fragment of an inhibitor with the protein binding pocket is computed in a pairwise manner as the sum of amino-acid residue/inhibitor interaction energies for a set of residues defining the pocket or subpocket.

To validate the $E_{EL,MTP}^{(10)}+E_{Das}$ approximation, here we compute several contributions to the second-order Møller–Plesset (MP2) interaction energy and assess their importance by evaluating correlation coefficients with experimentally determined inhibitory activities [20]. In these inhibitory activity models, we neglect the influence of binding free energy contributions such as entropy, desolvation energy and conformational adaptation of ligands and receptor upon binding. Our results suggest that this is a valid approximation when considering the relative binding free energies of a congeneric series of inhibitors that are expected to have similar binding modes. In addition, we examine various nonempirical representations of the dispersion term, to test the validity of the $E_{Das}$ approximation and the possibility of exchanging $E_{Das}$ with other dispersion corrections used with various DFT functionals. It should be noted that such corrections represent not only dispersion interactions but also other nonphysical deficiencies of DFT functionals [17].

In this study, we perform calculations for pteridine reductase 1 (PTR1), an enzyme involved in the pterin metabolism of trypanosomatid parasites [21, 22]. This enzyme, which is present in parasites but not humans, is a target for the design of inhibitors [20, 23–25] that disrupt the reduction of biopterin and folate in parasites and thus hinder their growth. In particular, PTR1 is an important enzyme in *Trypanosoma brucei* (*Tb*), which causes human African trypanosomiasis [26]. Different subpockets in the main binding site of *Tb*PTR1 have been explored in inhibitor discovery projects and it therefore provides a good system for assessing the applicability of the simple nonempirical model for fragment–based approaches to inhibitor design.

**Fig. 1:**
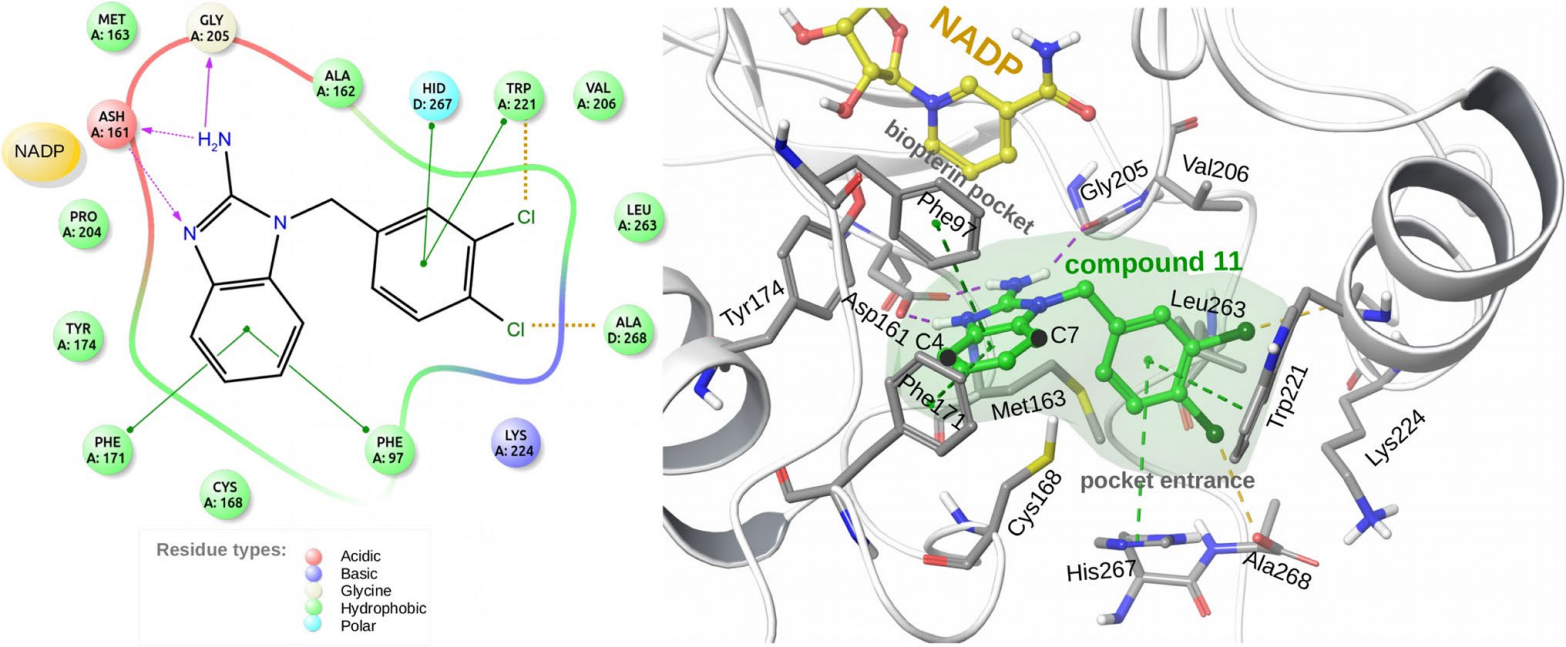
The crystal structure of *Tb*PTR1 in complex with compound 11 (PDB: 3GN2) showing the interactions made by the inhibitor in the binding site. (*left*) Interaction diagram of compound 11 with the *Tb*PTR1 protein pocket. A and D denote protein subunits in the *Tb*PTR1 homotetramer. (*right*) View of compound 11 (*green with green* semi-transparent surface contour) in the *Tb*PTR1 binding site, with residues within 3 Å of the ligand shown in stick representation and labeled. A fragment of the cofactor, nicotinamide adenine dinucleotide phosphate (NADP), is shown in *yellow*. The protein is rendered in cartoon representation. The substitution points (C4 and C7) in compound 11 are labeled. The edge-face $\pi$–$\pi$ interactions between the inhibitor and the protein are indicated by *green dashed lines*, while *purple and yellow dashed lines* denote hydrogen bonds and halogen bonds, respectively

In this work, we focus on the benzimidazol-2-amine series of potent, non-covalent, and reversible *Tb*PTR1 inhibitors that was developed by Mpamhanga et al. [23] and further extended by Spinks et al. [20], and for which apparent inhibitory activities ($K_i^{app}$; referred to in the following as ‘inhibitory activities’) against *Tb*PTR1 were measured. This series of compounds occupies the relatively hydrophobic *Tb*PTR1 subpockets adjacent to the binding site of the main enzyme substrate, biopterin (Fig. 1). Compound 11 from Ref. [20] (Fig. 1) is the parent compound for this inhibitor series [20, 23]. This compound adopts a well-defined binding mode in the crystal structure [23] (PDB code: 3GN2, see Fig. 1) stabilized by multiple hydrogen bonds, halogen bonds and stacking interactions. In particular, the N3 nitrogen of benzimidazole and the 2-amino group make hydrogen-bonds with the carboxylate group of the nearby Asp161 and the backbone carbonyl of Gly205 residue. On the other side of the binding pocket, the chlorines of the 3,4-dichlorophenyl moiety make halogen bonds with the backbone carbonyl group of Trp221 and the carboxylate group of the terminal Ala268 residue. The position of the scaffold of compound 11 is additionally stabilized by edge-face $\pi$–$\pi$ interactions (Fig. 1). Due to this extensive interaction pattern, we expect similar binding modes for the derivatives of compound 11. This assumption was used to model the *Tb*PTR1–inhibitor complexes, for which crystallographic structures were not available.

**Fig. 2:**
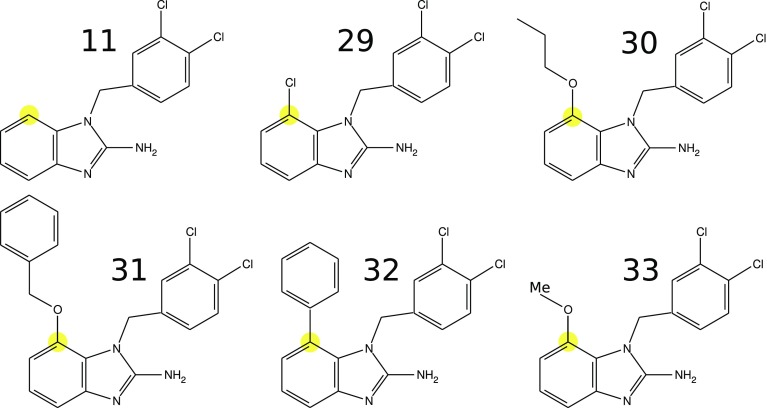
Chemical structures of the *C7* set of *Tb*PTR1 inhibitors [20]. The C7 position of the benzimidazole moiety is marked in *yellow*

**Fig. 3:**
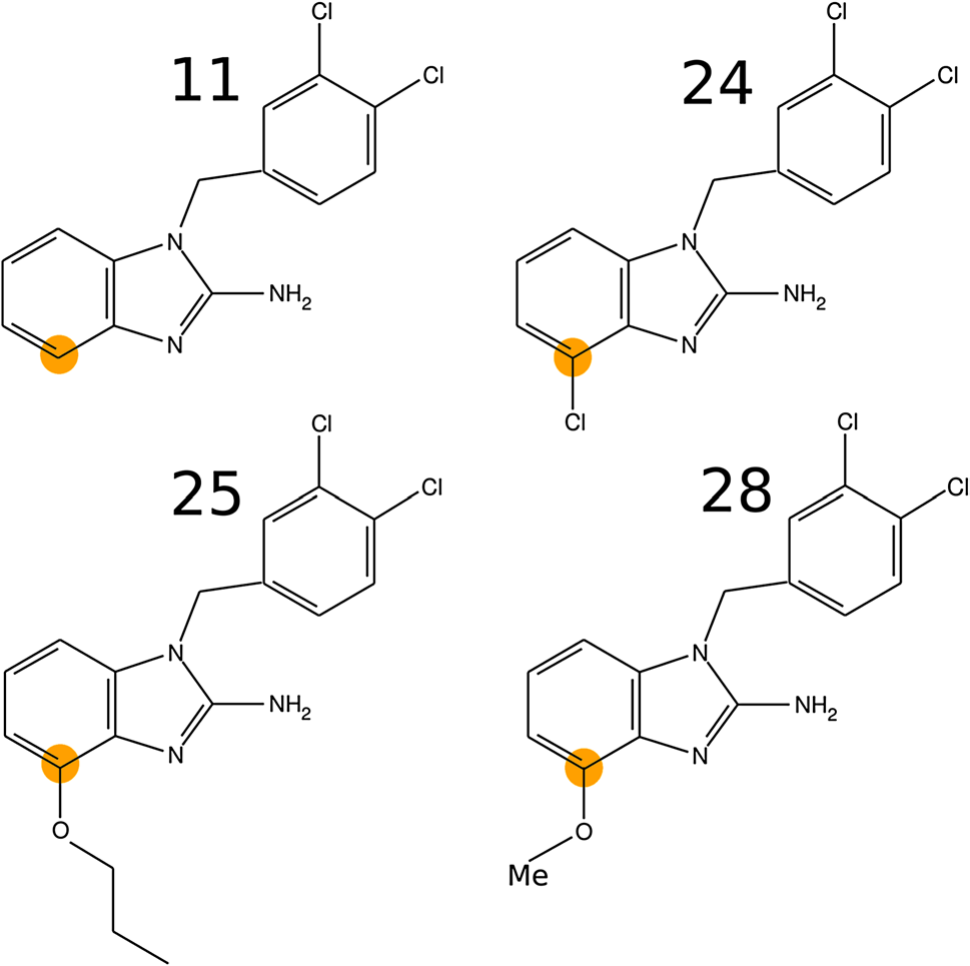
Chemical structures of the *C4* set of *Tb*PTR1 inhibitors [20]. The C4 position of the benzimidazole moiety is marked in *orange*

To evaluate the $E_{EL,MTP}^{(10)}+E_{Das}$ model for prediction of inhibitory activity, we first analyzed *Tb*PTR1 derivatives of compound 11 substituted at C7 of the benzimidazole scaffold, i.e. the compounds reported in Fig. 2 (which we refer to as the *C7* set). A similar analysis was previously performed for the docked covalent inhibitors of the FAAH enzyme [7]. The FAAH inhibitors were however modelled without knowledge of the crystallographically confirmed binding mode of the core scaffold, which probably introduced uncertainty into the results of the scoring model. Here, our first aim was therefore to test the performance of the $E_{EL,MTP}^{(10)}+E_{Das}$ model for another protein target, *Tb*PTR1, with an inhibitor series with a well-defined binding mode. Our second aim was to investigate whether the model is general and additive, which is assumed since the interaction energy is calculated as a sum of the pairwise residue/inhibitor contributions. Thus, we made a similar model based on the model for the *C7* set for the dataset of the C4–substituted compounds shown in Fig. 3 (which we refer to as the *C4* set), with substituents interacting predominantly with a different set of residues than the *C7* set. Our models for the *C4* and *C7* sets show transferability of the $E_{EL,MTP}^{(10)}+E_{Das}$ model, suggesting its applicability to fragment-based drug design approaches.

## Methods

### Preparation of the sets of compounds

Calculations were performed for (*i*) inhibitors from the *C7* set [20] (Fig. 2) and (*ii*) inhibitors from the *C4* set [20] (Fig. 3), which share a common parent scaffold: compound 11. The numbering of the inhibitors is adopted from Spinks et al. [20]. Of the reported inhibitors with substitutions at the C4 carbon of benzimidazole [20], two compounds, 26 and 27, were not included in the *C4* set due to their weak inhibitory activities and because their binding modes presumably differ from that characterizing the remaining inhibitors (as suggested by our docking simulations). Further assumptions underlying our approach, i.e., the representation of the receptor and ligand structures with models of limited size, preclude consideration of inhibitors with entirely different binding poses. Compound 28 (Fig. 3) was reported to be contaminated with 25% of compound 33 (Fig. 2), and the inhibitory activity was measured for a mixture [20]. Thus, we calculated the inhibitory activity of the pure compound 28 based on the equilibrium equations for competitive binding. As reported in the Supplementary material, Section 1, the computed value was $K_i^{app}=0.42~{\upmu}{\text{M}}$.

### Modeling of the protein–inhibitor complexes

The binding poses of the *C7*–set inhibitors were modelled in the *Tb*PTR1 binding pocket based on the crystallographic binding mode of compound 11 (PDB code 3GN2 [23]). Following the recommended protein preparation protocol [27], crystallographic water molecules were removed and the resulting protein–inhibitor complexes were minimized in Maestro [28] using Protein Preparation Wizard [29] with the default OPLS 2005 [30] force field, and the convergence criterion defined as the non-hydrogen atom RMSD = 0.3 Å. Optimal hydrogen bonding was determined with PROPKA [31–34], implemented in Maestro, at pH 6.0, i.e. the pH used for the measurements of inhibitory activities [20].

The modelling procedure was similar for the C4– and C7–substituted compounds. However, for the *C4* set, the modelling was more difficult because the *Tb*PTR1 subpocket accommodating the C4 benzimidazole substituents is smaller and more enclosed than that for the *C7* set. The binding pose of inhibitor 24 of the *C4* set was modelled as described above, but we also analyzed the unminimized protein–inhibitor complex. For compounds 25 and 28, the conformations of the top score poses from QM-polarized docking (implemented in Maestro software suite [28]) were taken for the aforementioned minimization and structure preparation protocol. The QM-polarized docking procedure consisted of the following steps: (*i*) Glide docking with XP (extra-precision) score [35, 36] and standard molecular mechanics OPLS 2005 force field electrostatic charges, (*ii*) recalculation of the semi-empirical Coulson’s electrostatic point charges for the docked ligands in the protein surrounding, and (*iii*) redocking of the inhibitors with the electrostatic charges calculated in step (*ii*).

### Definition of the ligand fragments and *Tb*PTR1 binding subpockets

**Fig. 4:**
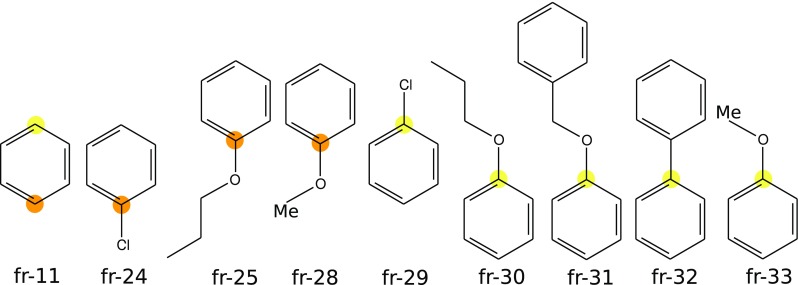
Fragments of the *Tb*PTR1 inhibitors defined for calculations. Carbon atoms corresponding to the C7 and C4 positions of benzimidazole in the full inhibitor structures are marked in *yellow* and *orange*, respectively

Since the binding mode of the common core of the inhibitors considered (i.e., compound 11) is well defined and likely to be positioned similarly for all inhibitors, its contribution to the observed binding affinity differences is most probably negligible. Therefore, to decrease the computational cost for the ab initio interaction energy calculations, the inhibitor structures were truncated to the fragments shown in Fig. 4 (named with a ‘fr-’ prefix). All the calculations refer to the inhibitor fragments shown in Fig. 4.

**Fig. 5:**
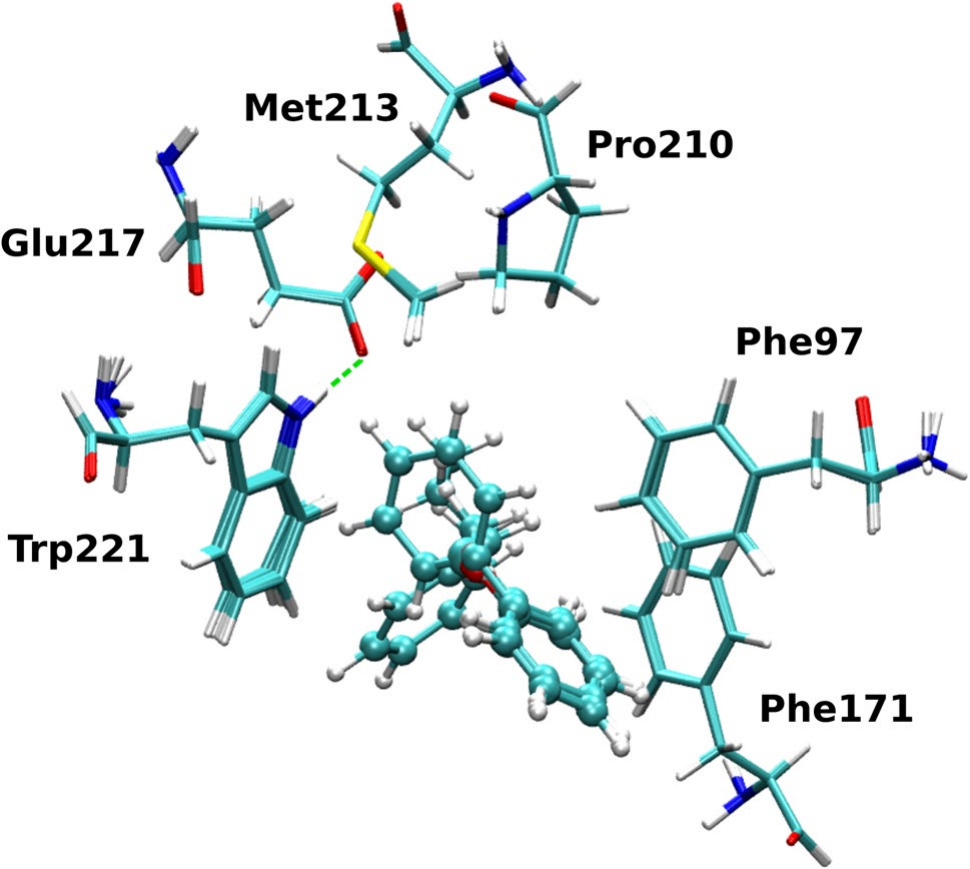
The *C7* subpocket and ligand fragments. Superimposed structures of the complexes of *Tb*PTR1 with the *C7* inhibitor fragments showing the surrounding residues included in the calculations. The hydrogen bond between Glu217 and Trp221 is shown by a *green dashed line*

**Fig. 6:**
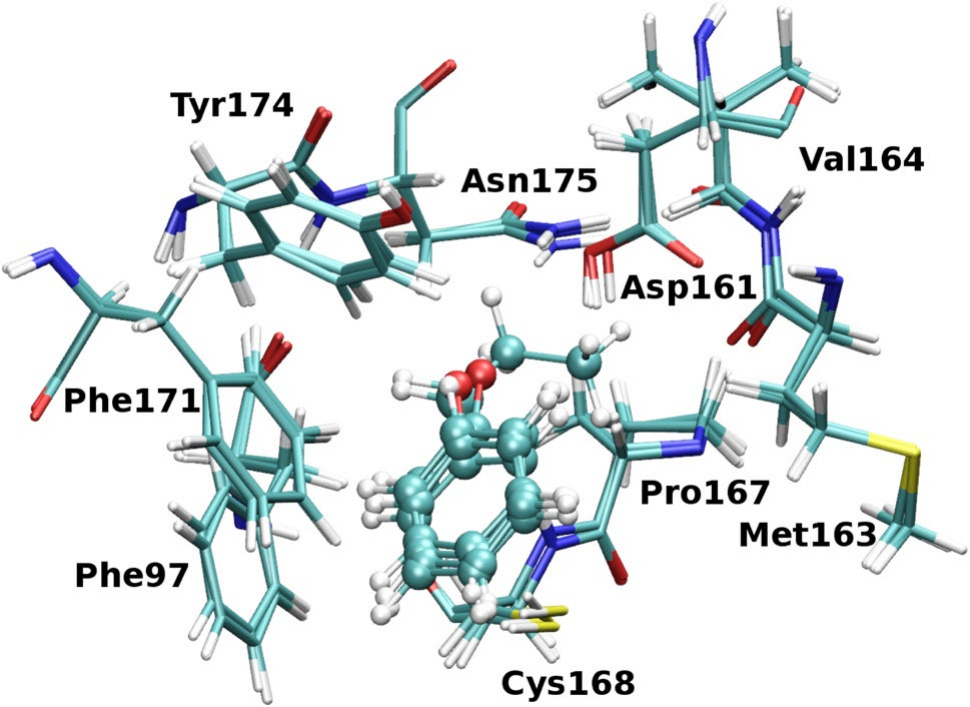
The *C4* subpocket and ligand fragments. Superimposed structures of the complexes of *Tb*PTR1 with the *C4* inhibitor fragments showing the surrounding residues included in the calculations

The *Tb*PTR1 *C7* binding subpocket consisted of residues from the first shell surrounding the *C7* inhibitor fragments, see Fig. 5. Due to the potential flexibility of Cys168 and the limited ability of the restrained optimization protocol to account for more extensive conformational changes, Cys168 was excluded from the subpocket definition. Finally, the following residues were present in the *C7* receptor model used for evaluating the interaction energy: Phe97, Phe171, Pro210, Met213, Glu217 and Trp221. Since Glu217 was hydrogen-bonded to Trp221, this residue was included in the calculations as a part of a Glu217–Trp221 dimer. The broken bonds arising from cutting the residues out of the protein scaffold were capped with hydrogen atoms optimized with Maestro using the protocol described above. The *C4* system consisted of the following residues: Phe97, Asp161, Met163, Val164, Pro167, Cys168, Phe171, Tyr174, and Asn175, see Fig. 6. In this subsystem, in contrast to *C7*, Cys168 was included, because the rigid backbone of Cys is relatively close to the *C4* substitution site, whereas the flexible side chain is not in direct contact (sulphur atom of Cys168 is located about 4.05 Å from carbon C4). Furthermore, in the main set of results, Asp161 was treated as protonated and the protonation of Asp161 at acidic pH is consistent with the catalytic mechanism of PTR1, as suggested by Gourley et al. [37]. Notably, the systems with protonated Asp161 display significantly better correlation of the energy contributions with the $p\text {K}_{\text {i}}^{app}$ values than those with unprotonated Asp161 (data shown in Tables S5 and S6 in the Supplementary Material).

### Ab initio energy decomposition scheme

Hybrid variation-perturbation theory (HVPT) [38, 39] was applied to partition the interaction energy calculated at the Møller–Plesset second-order level of theory, $E_{MP2}$, into the following contributions characterized by increasing computational cost, as indicated by *O*(*X*) scaling (where *N* and *A* stand for the number of atomic orbitals and atoms, respectively):1
$E_{EL,MTP}^{(10)}$ refers to the electrostatic multipole component estimated from an atomic multipole expansion [40] (see the Supplementary Material for a detailed description of the interaction energy terms discussed herein). $E_{EL,PEN}^{(10)}$ is the electrostatic penetration energy, calculated from the following expression: $E_{EL,PEN}^{(10)}$ = $E_{EL}^{(10)}$ − $E_{EL,MTP}^{(10)}$, where $E_{EL}^{(10)}$ represents the first-order electrostatic energy. The first-order exchange energy $E_{EX}^{(10)}$ term in Eq. 1 is calculated from the first-order Heitler–London energy, $E^{(10)}$: $E_{EX}^{(10)}$ = $E^{(10)}$ − $E_{EL}^{(10)}$. The higher-order delocalization energy, $E_{DEL}^{(R0)}$, is calculated as: $E_{DEL}^{(R0)}$ = $E_{SCF}$ − $E^{(10)}$, where $E_{SCF}$ is the counterpoise-corrected self-consistent field variational energy. The correlation term is defined as: $E_{CORR}^{(2)}$ = $E_{MP2}$ − $E_{SCF}$. The $E_{EL,MTP}^{(10)}$ and $E_{CORR}^{(2)}$ contributions are considered long-range energy terms, i.e. they vary with the intermolecular distance *R* as $R^{-n}$ ($n \in \mathbb {N}$), whereas $E_{EL,PEN}^{(10)}$, $E_{EX}^{(10)}$ and $E_{DEL}^{(R0)}$ are short-range terms, i.e. they vary as $exp^{-\gamma R}$ with $R, \gamma>0$ (see Eq. 1).

### Ab initio interaction energy calculations

The interaction energy between each residue (a single residue monomer or the Glu217–Trp221/Met163–Val164 dimers) and each inhibitor fragment was calculated with a modified version [39] of the GAMESS program [41] using the 6-311G(d) [42, 43] basis set with diffuse functions on the s and p orbitals of the chlorine atoms [44, 45]. This approach was chosen rather than using a full 6-311++G(2d,2p) basis set to save computational time and because we did not notice major qualitative differences in the results (a comparison is given in the Supplementary material, Table S1). A counterpoise correction was applied to avoid basis set superposition error [46].

Multipole electrostatic energy terms were calculated using the Cumulative Atomic Multipole Moments (CAMM) approach [40, 47] implemented in GAMESS with the expansion truncated at the $R^{-3}$ term. Exponential truncation of the CAMM expansion at the $(1/R)^{n}$ term seems to limit the disruptive effect of diffuse functions on higher rank multipoles and yields the best results for $n=3$, i.e. including the atomic dipole–dipole and monopole–quadrupole terms.

In addition, three dispersion energy models were tested and compared as regards their suitability for this application, namely: (*i*) as a reference, the $E_{CORR}^{(2)}$ term from the HVPT energy decomposition scheme, (*ii*) the $E_{Das}$ function [17, 19] fitted to the SAPT [18] results, and (*iii*) the D3 correction [48] to dispersionless—DFT with Becke–Johnson damping [49] applied. The latter was calculated with standalone DFT-D3 program (version 3.1. Rev 1) [48, 49], using three different functionals, namely the hybrid functionals PBE0 [50, 51] and B3LYP [52], and the exchange functional TPSS [53]. D3 is defined as a dispersion correction being the sum of two- and three-body contributions to the dispersion energy [48].

### Data analysis

Each scoring model considered was compared with the experimental inhibitory activities ($p\text {K}_{\text {i}}^{app}$) by computing Pearson correlation coefficients (R) and the success rate of prediction of relative binding affinities ($N_{pred}$). The latter is defined as the  percentage of concordant inhibitor pairs with relative stability of the same sign as in the reference experimentally measured activities, evaluated among all pairs of the inhibitor set (*C4* or *C7*) [54]. Note that when Pearson correlation coefficients between the calculated interaction energies in our model and the experimental inhibitory activities are *negative* (trends of the aforementioned data sets are opposite), these values are said to *correlate*, as lower values of energy should ideally accompany increasing inhibitory activity. For the inverse relationship associated with a *positive* Pearson correlation coefficient, we use the term “anticorrelation”. The XP (extra-precision) scoring in the Glide program [35, 36, 55], with the “score-in-place” option, was applied for empirical scoring of the fragments used for the ab initio calculations. The score function is expressed as follows:

2 $$\begin{aligned} {XP~GlideScore} = E_{coul} + E_{vdW} + E_{bind} + E_{penalty} \end{aligned}$$

$E_{coul}$ and $E_{vdW}$ are electrostatic and van der Waals terms, respectively, $E_{penalty}$ includes a desolvation penalty and a ligand strain energy which are unfavorable for binding, while $E_{bind}$ is composed of favourable terms similar to those in the ChemScore function [56]. The $E_{bind}$ terms of the XP GlideScore additionally include: (*i*) a model of hydrophobic interactions, which takes into account ligand hydrophobic enclosure, and (*ii*) an improved model of hydrogen bond interactions [36]. The above scoring function was tested on 198 protein–ligand complexes, resulting in binding free energy RMSDs of 2.26 and 1.73 kcal mol^−1^ over all and selected well-docked ligands, respectively, as reported by Friesner et al. [36].

## Results and discussion

### The $E_{EL}^{(10)}$ and $E_{MP2}$ interaction energies correlate with $p\text {K}_{\text {i}}^{app}$ values of the *C7* set

The total interaction energy values calculated at the consecutive levels of theory are shown in Table 1. Moreover, the relationship between these interaction energies and exchange, delocalization and correlation contributions is depicted in Fig. S1 in the Supplementary Material. The corresponding pairwise interaction energy values for inhibitor–residue pairs and the numerical values of exchange and delocalization energy components are presented in the Supplementary material (Tables S2, S3, respectively).

**Table 1:** The total interaction energy at consecutive levels of theory, and energies for the $E_{EL,MTP}^{(10)}+E_{Das}$ and $E_{EL,MTP}^{(10)}+E_{CORR}^{(2)}$ models for the *C7* set of inhibitors

Inhibitor	$p\text {K}_{\text {i}}^{app}$ ^a^	$E_{EL,MTP}^{(10)}$	$E_{EL}^{(10)}$	$E^{(10)}$	$E_{SCF}$	$E_{MP2}$	$E_{EL,MTP}^{(10)}+E_{Das}$	$E_{EL,MTP}^{(10)} + E_{CORR}^{(2)}$
fr-32	8.2	−4.4	−8.3	10.8	7.0	−9.5	−25.8	−20.9
fr-30	7.3	−3.4	−6.9	7.6	4.5	−8.8	−21.9	−16.7
fr-31	7.0	−0.2	−7.0	12.2	8.9	−7.9	−20.2	−16.9
fr-29	6.3	−1.3	−3.0	6.2	4.6	−4.0	−12.2	−9.8
fr-33	6.2	−3.0	−5.9	5.3	3.3	−6.4	−15.5	−12.7
fr-11	6.1	−2.5	−4.6	5.1	3.6	−4.1	−12.4	−10.1
R$^{\text{b}}$		−0.48	−0.84	0.76	0.62	−0.89	−0.96	−0.95
$N_{pred}^{\text{c}}$		66.7	80.0	13.3	26.7	86.7	86.7	80.0

In units of kcal mol^−1^

$^{\text{a}}$Experimental $p\text {K}_{\text {i}}^{app}$values are taken from Ref. [20]

$^{\text{b}}$Pearson correlation coefficient between the calculated energy and the experimental inhibitory activity

$^{\text{c}}$Percentage of concordant pairs (%)

Table 1 shows that the $E_{MP2}$ interaction energy significantly correlates with the experimentally determined inhibitory activities (R = $-0.89$). Apart from fr-29, the $E_{MP2}$ values reflect the ranking of the inhibitors established experimentally. Furthermore, the $E_{EL}^{(10)}$ values correlate with the experimental data (R = $-0.84$), but with a lower $N_{pred}$ value than for $E_{MP2}$ (80 vs. 86.7%, respectively).

For the fragments of three least potent inhibitors of the *C7* set, fr-29, fr-33, and fr-11, the differences in $p\text {K}_{\text {i}}$ of about 0.1 (corresponding to an approximately 0.1 kcal mol^−1^ difference in binding free energy [57]), cannot be expected to be reproduced computationally, as they exceed the accuracy of most quantum chemical calculations, and, likely, of the experiments (measurement errors are, however, not explicitly provided in Refs. [20, 23]). Accordingly, the computed binding energies of fr-29, fr-33, and fr-11 ligands would be expected to be similar. It can be seen in Table 1 that fr-29 and fr-11, but not fr-33, have similar $E_{MP2}$ interaction energy values. Actually, the $E_{MP2}$ binding energies of the *C7* set inhibitors with an oxygen atom at the C7 position (i.e., compounds 30, 31, and 33) appear to be slightly overestimated (see Fig. S2 in the Supplementary material) whereas, the inhibitors with a hydrophobic C7 substituent have underestimated values of $E_{MP2}$ interaction energy. Notably, the C7 substituents are in direct contact with the methylene linker of the common scaffold depicted in Fig. 2 (e.g., the distance between the C7 chlorine and the closest hydrogen atom of the adjacent methyl group in compound 29 is 2.7 Å). Considering that this particular part of the inhibitor was not included in binding energy analysis, the observed under- and overestimation of interaction energy could be associated with intramolecular interactions that are not accounted for by the model. Despite these omissions, the overall correlation with experimental inhibitory activity is satisfactory.

On the other hand, both $E^{(10)}$ and $E_{SCF}$ anti-correlate with inhibitory activities, i.e., these energies tend to indicate greater repulsion for the compounds with higher inhibitory activity. Positive values of the $E^{(10)}$ and $E_{SCF}$ energies result from the repulsive exchange contribution, $E_{EX}^{(10)}$ (Table S3 in the Supplementary material). The repulsion is stronger for more potent inhibitors, since the molecular fragments that interact strongly are also more likely to experience stronger exchange effects [54, 58]. For example, for fr-31, the $E^{(10)}$ and $E_{SCF}$ interaction energy terms appear to be exceptionally high (i.e., unfavorable) due to the incorporation of short-range exchange contribution. The short-range effects are exponentially dependent on the interatomic distance, so any minor structural defect (e.g., artificial shortening of the intermolecular distance due to the basis set superposition error or the difference between the crystal structure and the gas phase or the force field non-transferability) results in significant errors in the energy values. Therefore, $E^{(10)}$ and $E_{SCF}$ are vulnerable to structural deficiencies in the model [7, 58], and are not really applicable for studying crystal structure-based models of the protein–ligand complexes.

Although the delocalization term, $E_{DEL}^{(R0)}$, accounted for at the Hartree–Fock level of theory is characterized by a correlation coefficient R = $-0.94$ (see the Supplementary material, Table S3), it is still insufficient to overcome the inverse inhibitory ranking due to the $E_{EX}^{(10)}$ contribution (Fig. S1 in the Supplementary material). Only the $E_{CORR}^{(2)}$ contribution to the $E_{MP2}$ energy restores the proper ranking of the inhibitory activities (Table 1). As demonstrated in Fig. S1 in the Supplementary material, both the $E_{DEL}^{(R0)}$ and $E_{CORR}^{(2)}$ terms approximately cancel out the repulsive characteristics of the exchange component, but $E_{CORR}^{(2)}$ is the major contribution out of these two.

The above analysis is in line with our previous findings for FAAH inhibitors [7] that omitting the contributions arising from short-range interactions (i.e., considering only the multipole electrostatic and correlation energy components) results in a much better model for predicting the inhibitory activity [7].

### The $E_{Das}$ approximation predicts inhibitory activities of the *C7*–set inhibitors as well as the correlation energy

The results shown in Table 2 indicate that, for the *C7*–set inhibitors, the dominant dispersion contribution could even serve as a standalone scoring method, with R = $-0.86$ for $E_{CORR}^{(2)}$. Furthermore, for the *C7* inhibitor set, the $E_{Das}$ approximation correlates with the inhibitory activities as well as $E_{CORR}^{(2)}$ (R = $-0.92$), thereby confirming the validity of the $E_{Das}$ approximation. A significant correlation is obtained for the $E_{CORR}^{(2)}$ and $E_{Das}$ energies because the *C7*–set of inhibitor fragments consists of largely hydrophobic substituents targeting a relatively hydrophobic *Tb*PTR1 subpocket, and thus dispersive interactions play a major role in binding.

**Table 2:** $E_{Das}$ function and the D3 dispersion energy approximations evaluated for the *C7*–set inhibitor fragments

Inhibitor	$p\text {K}_{\text {i}}^{app}$ ^a^	$E_{CORR}^{(2)}$	$E_{Das}$	D3$_{PBE0}$	D3$_{B3LYP}$	D3$_{TPSS}$
fr-32	8.2	−16.5	−21.4	−12.3	−17.6	−15.7
fr-30	7.3	−13.3	−18.5	−10.9	−15.5	−13.8
fr-31	7.0	−16.8	−20.1	−12.2	−17.1	−15.4
fr-29	6.3	−8.5	−10.9	−6.5	−9.2	−8.2
fr-33	6.2	−9.7	−12.5	−7.5	−10.5	−9.4
fr-11	6.1	−7.6	−9.9	−5.7	−8.2	−7.3
R$_{CORR}$ ^b^			0.98	0.99	0.99	0.99
R^c^		−0.86	−0.92	−0.89	−0.90	−0.89
N$_{pred}$ ^d^		66.7	86.7	86.7	86.7	86.7

In units of kcal mol^−1^

$^{\text{a}}$Experimental affinity values are taken from Ref. [20]

$^{\text{b}}$Pearson correlation coefficient between the dispersion approximation and the correlation energy $E_{CORR}^{(2)}$

$^{\text{c}}$Pearson correlation coefficient between the calculated dispersion approximation and the experimental inhibitory activity taken from Ref. [20]

$^{\text{d}}$Percentage of concordant pairs (%)

Nonetheless, a universal scoring model should also account for the electrostatic contribution, so that it is appropriate for noncovalent complexes with intermolecular forces of either dispersive or electrostatic nature (or a mixture of both). Upon including the electrostatic multipole term, the scoring abilities of the proposed model further improve for the *C7* inhibitor set (Table 1): for $E_{EL,MTP}^{(10)}+E_{Das}$ and $E_{EL,MTP}^{(10)}$ + $E_{CORR}^{(2)}$ models the correlation coefficients are equal to $-0.96$ and $-0.95$, respectively. Furthermore, the $E_{EL,MTP}^{(10)}+E_{Das}$ model correctly predicted 86.7% of concordant pairs, i.e. as many as for the $E_{MP2}$ level. Note that for the $E_{EL,MTP}^{(10)}+E_{Das}$ model describing *Tb*PTR1 inhibition, the correlation is distinctly better than for the inhibitors of the FAAH enzyme (*R* = $-0.67$) [7]. This difference might be due to the higher flexibility of the FAAH inhibitor series, which may cause problems in determining the correct binding pose even by the most accurate docking procedures.

### Comparison of dispersion models: alternatives to the $E_{Das}$ function

In the work presented herein, we additionally tested the D3 correction to dispersionless—DFT developed by Grimme et al. [48], calculated for the following functionals: PBE0, B3LYP, and TPSS. Our objective was to check how the simple $E_{Das}$ approximation is related to the widely tested D3 correction. The performance of the dispersion approximations for the *C7* set is compared in Table 2.

All the dispersion approximations correlate well with the $E_{CORR}^{(2)}$ results. Considering the agreement with the experimental inhibitory activities, the dispersion energies calculated from the D3 corrections perform comparably well with the other methods, with a correlation coefficient *R* of about $-0.90$ for all the three tested functionals, whereas for $E_{Das}$ and $E_{CORR}^{(2)}$ the values of *R* are equal to $-0.92$ and $-0.86$, respectively. While each dispersion approximation considered here performs remarkably well, the dispersion model proposed by Pernal et al. [17] is fitted to the SAPT results, whereas the results obtained with D3 corrections might, in general, depend on the applied functional. Accordingly, the choice of certain functional might not be obvious if structure–activity relationships are to be predicted for novel receptor–ligand complexes not characterized experimentally, wherein prior testing of the performance of a given method cannot be carried out.

### Comparison of the $E_{EL,MTP}^{(10)}+E_{Das}$ model with empirical scoring

**Fig. 7:**
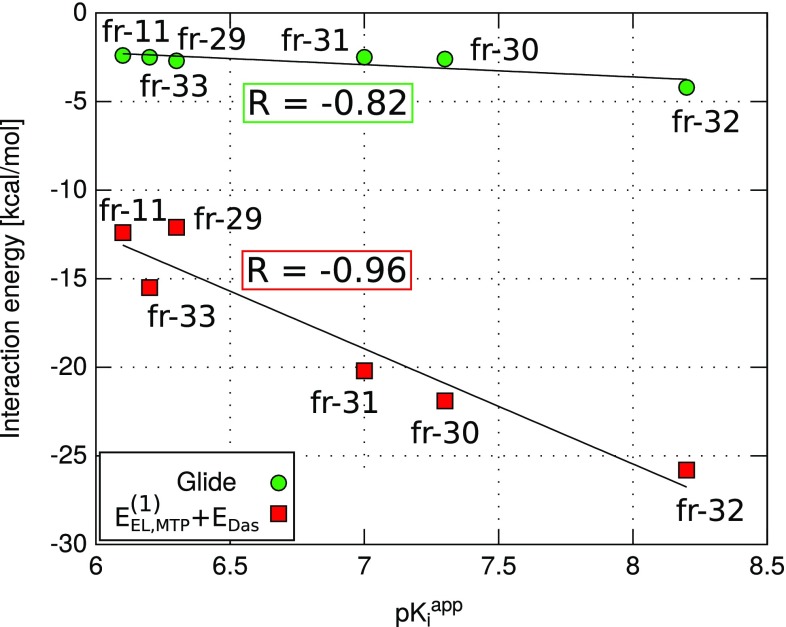
Interaction energies of the  *C7* set of  *Tb*PTR1 inhibitor fragments computed with nonempirical and empirical scoring approaches versus experimental inhibitory activities taken from Ref. [20]

Protein–ligand interactions are routinely evaluated with computationally inexpensive empirical scoring approaches [59]. These rely on empirical parameters that are typically derived using a diverse training set. Although, in principle, generally applicable, such scoring functions have been shown to perform successfully in some applications and to fail in others [1, 60]. In Fig. 7, the nonempirical $E_{EL,MTP}^{(10)}+E_{Das}$ scoring method is compared with the Glide  XP scoring function [35]; the corresponding values are given in the Supplementary material (Table S4). Notably, the predictive capabilities of the $E_{EL,MTP}^{(10)}+E_{Das}$ model, tested on the *C7* set of inhibitor fragments, outperform the empirical scoring function, with an *R* correlation coefficient of $-0.96$ compared to $-0.82$ value obtained with Glide XP.

An important advantage of the $E_{EL,MTP}^{(10)}+E_{Das}$ model is that it is fully based on well-defined long-range interaction energy components obtained from first principles ab initio calculations and does not contain any empirical parameters. Moreover, the method is as computationally affordable as commonly used empirical scoring functions, scaling as $O(A^2)$ [7]. Overall, these results suggest that more accurate, nonempirical ways of assessing the protein–ligand interactions might be advantageous for the design of novel inhibitors, and that a gain in the quality of the results can be achieved with little computational effort.

### Sensitivity of the $E_{EL,MTP}^{(10)}+E_{Das}$ and $E_{MP2}$ models to the inhibitor–receptor modelling procedure—the case of compound 24 in the *C4* set of inhibitors

We performed calculations for the *C4* set of inhibitor fragments, targeting another subpocket of *Tb*PTR1. Table 3 shows the interaction energies calculated at the different levels of theory, together with the $E_{EL,MTP}^{(10)}$ + $E_{CORR}^{(2)}$ and $E_{EL,MTP}^{(10)}+E_{Das}$ models. The corresponding interaction energy values for each residue–inhibitor fragment pair are presented in the Supplementary Material (Table S6).

**Table 3:** The total interaction energy at consecutive levels of theory, and energies for the $E_{EL,MTP}^{(10)}+E_{Das}$ and $E_{EL,MTP}^{(10)}+E_{CORR}^{(2)}$ models for the *C4* set of inhibitors

Inhibitor	$p\text {K}_{\text {i}}^{app}$ ^a^	$E_{EL,MTP}^{(10)}$	$E_{EL}^{(10)}$	$E^{(10)}$	$E_{SCF}$	$E_{MP2}$	$E_{EL,MTP}^{(10)}+E_{Das}$	$E_{EL,MTP}^{(10)}+E_{CORR}^{(2)}$
fr-25	6.5	−1.7	−16.9	32.9	24.6	−4.4	−48.8	−30.7
fr-28	6.4	−0.1	−11.7	21.4	16.0	−5.8	−33.4	−22.0
fr-11	6.1	1.0	−9.5	19.5	14.8	−2.4	−22.7	−16.2
fr-24	5.6	−0.2	−10.4	18.7	13.9	−6.0	−29.8	−20.1
R$^{\text{b}}$		−0.44	−0.66	0.70	0.71	0.21	-0.59	-0.59
${N_{pred}}^{\text{c}}$		66.7	83.3	0.0	0.0	33.3	83.3	83.3
fr-24$^*$	5.6	−2.1	−17.7	27.1	19.9	−1.7	−36.0	−23.7
R$^{*{\text{b}}}$		0.27	0.28	0.14	0.17	−0.85	−0.35	−0.33
${N^{*}_{pred}}^{\text{c}}$		50.0	50.0	33.3	33.3	83.3	66.7	66.7

In units of kcal mol^−1^

The data for the unminimized compound 24 complex are marked by *

$^{\text{a}}$Experimental affinity values are taken from Ref. [20]

$^{\text{b}}$Pearson correlation coefficient between the calculated energy and the experimental inhibitory activity

$^{\text{c}}$Percentage of concordant pairs (%)

Since modeling of the *C4* set of inhibitor–protein complexes was not as straightforward as for the *C7* set, we analyzed the influence of the modeling procedure, in particular the classical force field minimization of the *Tb*PTR1–inhibitor complex, on the computed energy values for one inhibitor, compound 24. In this case, we suspected that the classical force field description [30], which models atoms as spheres with point charges, might incorrectly treat the interactions of 4-Cl substituent with the side chain of Asn175. Due to the complex electronic structure of halogens and the resultant $\sigma$-hole phenomenon, the chlorine may form the so-called halogen bond, which is poorly described in commonly-used force fields [61].

We observed that classical minimization distinctly changes coordinates of this system, with the distance between 4-Cl of compound 24 and amide N of Asn175 decreasing from 5.0 to 3.9 Å (see Fig. S3 in the Supplementary material). For the *minimized* compound 24 complex, the exchange energy $E_{EX}^{(10)}$ is much lower than for the *unminimized* complex (29.2 vs. 44.7 kcal mol^−1^, see Table S7 in the Supplementary material). This difference suggests that minimization reduces some steric clashes in the complex. Furthermore, with *minimized* compound 24, $E_{EX}^{(10)}$ anti-correlates with $p\text {K}_{\text {i}}^{app}$ for the four *C4* compounds, whereas with the *unminimized* compound 24 complex, no such anti-correlation is observed ($R=0.69$ vs. $-0.03$, respectively, Table S7). This is likely the reason why $E_{MP2}$ does not correlate with $p\text {K}_{\text {i}}^{app}$ for the *C4* set with *minimized* inhibitor 24 ($R=0.21$, Table 3), whereas it correlates for the *unminimized* compound 24 ($R=-0.85$). On the other hand, the $E_{EL,MTP}^{(10)}+E_{Das}$ model (which does not include $E_{EX}^{(10)}$) shows the expected inverse relation with $p\text {K}_{\text {i}}^{app}$ for both the *minimized* ($R = -0.59$) and the *unminimized* ($R=-0.35$) inhibitor 24 (Table 3). Thus, the results suggest that the $E_{EL,MTP}^{(10)}+E_{Das}$ model is more robust than the $E_{MP2}$–based model to inaccuracies in the models of the inhibitor–receptor complexes.

### Limitations of the $E_{EL,MTP}^{(10)}+E_{Das}$ model due to omission of the exchange energy

For the *C4* set, the correlation of $E_{EL,MTP}^{(10)}+E_{Das}$ with $p\text {K}_{\text {i}}^{app}$ is not as good as for the *C7* set (compare Tables 1,  3). This is likely due to the lower number of inhibitors and the narrow range of $p\text {K}_{\text {i}}^{app}$ values in this set. However, if we investigate how well the $E_{EL,MTP}^{(10)}+E_{Das}$ model approximates the reference $E_{MP2}$ energy, omitting compound 25 significantly increases the correlation between $E_{MP2}$ and $E_{EL,MTP}^{(10)}+E_{Das}$, which is 0.27 for all inhibitors and 0.93 when compound 25 is excluded from the C4 set (see Table S8 in the Supplementary material). Notably, $E_{EX}^{(10)}$ of compound 25 seems to be overestimated (unfavourable for binding), while $E_{CORR}^{(2)}$ (and $E_{Das}$) are too favourable (see Table S7 in the Supplementary material). For this compound, even the standard docking procedure shows that the side chain makes steric clashes, and these contacts could not be properly relaxed by energy minimization procedure (see Fig. S4 in the Supplementary Material). The omission of the $E_{EX}^{(10)}$ energy, and thereby the effects of short-range repulsion, in the $E_{EL,MTP}^{(10)}+E_{Das}$ model mean that it should not be applied when there are significant ligand–receptor clashes, such as observed for compound 25. This problem can be detected at the early stage of modeling/docking prior to the QM calculations. Since the $E_{EL,MTP}^{(10)}+E_{Das}$ model does not properly capture the interactions of inhibitor 25, we omitted this compound in further analysis.

### Putting the *C7* and *C4* models together

**Table 4:** Inhibitory activities $p\text {K}_{\text {i}}^{app}$ for the *C4* set predicted with the best fitting equations obtained from the $E_{EL,MTP}^{(10)}+E_{Das}$ and $E_{MP2}$ models

Inh.	Exp.^a^	$E_{EL,MTP}^{(10)}+E_{Das}$		*E*_*MP2*_	
		*C4*	C4*	*C4*	C4*
fr-28	6.4	6.8	6.4	6.5	7.0
fr-11	6.1	5.1	4.8	5.1	5.7
fr-24	5.6	6.2	6.9	6.5	5.4
RMSE$^b$		0.7	1.0	0.8	0.4

The model has the same gradient $\alpha$ as the corresponding *C7* model ($E = \alpha \cdot pK_i^{app} + \beta$)

The data sets with the unminimized compound 24 complex are marked by *

See model parameters in Table S9 in the Supplementary material

$^{\text{a}}$Experimental $p\text {K}_{\text {i}}^{app}$ values are taken from Ref. [20]

$^{\text{b}}$Root mean square error of $p\text {K}_{\text {i}}^{app}$ prediction

**Fig. 8:**
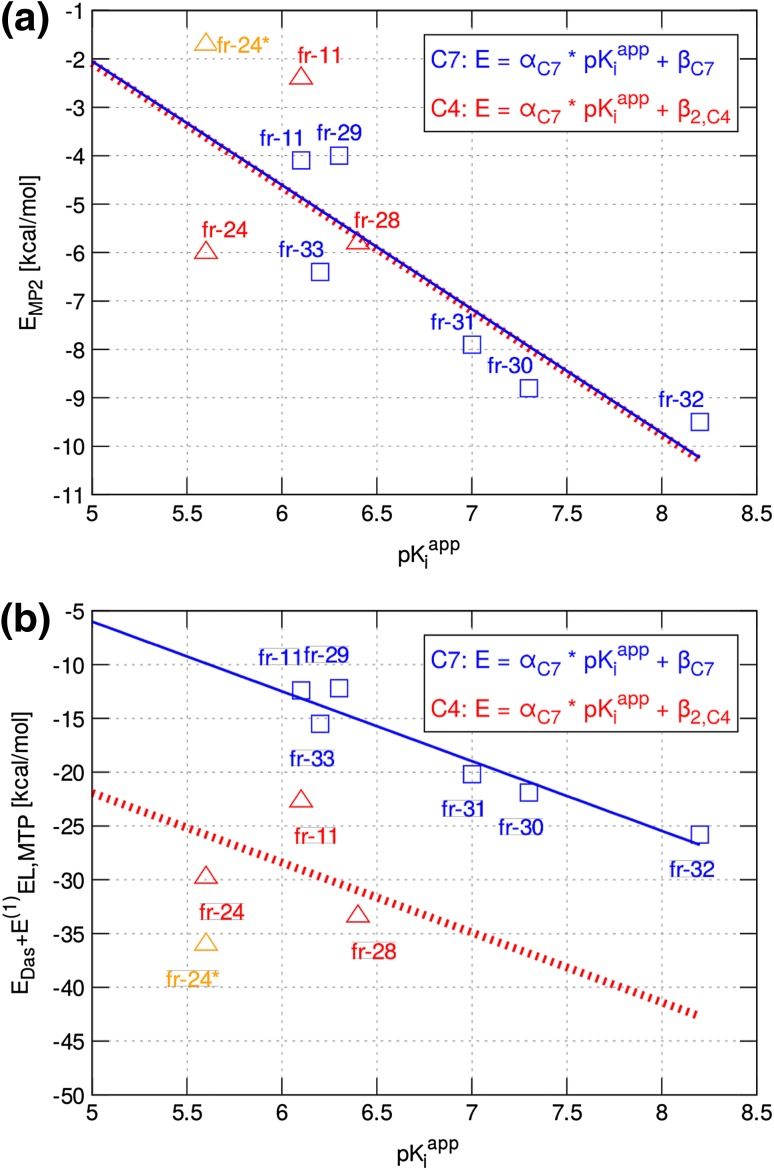
The $E_{MP2}$ (**a**) and $E_{EL,MTP}^{(10)}+E_{Das}$ (**b**) interaction energies versus experimental inhibitory activities and linear regression models for the *C4* and *C7* inhibitor sets. The same $\alpha$ value is used for the *C7* and *C4* models. Parameters of the models are presented in Table S9 in the Supplementary Material and $p\text {K}_{\text {i}}^{app}$ prediction results are given in Table 4. The data for the unminimized compound 24 (fr-24$^{*}$) are also shown (in *orange*)

Analysis of correlations for the *C4*–set models should be treated with caution since the dataset (excluding inhibitor 25) consists of only three inhibitors with a very narrow $p\text {K}_{\text {i}}^{app}$ range. Therefore, we did not make correlation models for these points alone. Instead, we fitted these data points to the *C7*–set $E_{EL,MTP}^{(10)}+E_{Das}$ and $E_{MP2}$ models, assuming per-residue additivity of the models (see explanation in the Supplementary material), which requires that $\alpha$ parameter in the $E = \alpha \cdot pK_i^{app} + \beta$ models for the *C7* system was kept constant and the $\beta$ parameter was fit by minimizing the deviation of the three *C4* experimental $p\text {K}_{\text {i}}^{app}$ values. The use of different $\beta$ values for different subpockets reflects the fact that there is a different energetic contribution to binding for each substituent position but that this contribution can be assumed constant for all compounds with substituents at a given position. Fig. 8 shows the fitted $E_{MP2}$ and $E_{EL,MTP}^{(10)}+E_{Das}$ models for the *C7* and the *C4* set. It can be seen in Fig. 8 that, for both inhibitor sets, predictions of the regression models are generally worse for the weaker binders, which form less specific and less stable interactions, and are thus more challenging to model.

The $E_{MP2}$ models for the *C7* set and the *C4* or C4* set (for unminimized inhibitor 24) are remarkably consistent (see Fig. 8a; Table 4). This is confirmed by low RMS errors in $p\text {K}_{\text {i}}^{app}$ values (0.8 and 0.4 for the *C4* and C4* sets, respectively; Table 4). Thus, the $E_{MP2}$ model seems to be transferable between the *C4* and *C7* pockets. The $E_{EL,MTP}^{(10)}+E_{Das}$ energy performs overall worse in predicting $p\text {K}_{\text {i}}^{app}$values than $E_{MP2}$ (Table 4), although it also shows reasonable transferability of the *C7* model to the *C4* subpocket (Fig. 8b; Table 4). The subpockets feature differing physico-chemical properties (*C7*—more hydrophobic, *C4*—more hydrophilic; compare Figs. 2, 3), but similar hydrophobic character of the substituents may facilitate the transferability of the models between subpockets, which should be further tested for ligands with more diverse properties.

## Concluding remarks

We have conducted ab initio calculations of the interaction energies between a series of inhibitor fragments and binding subpockets of the *Tb*PTR1 enzyme. The common scaffold for this inhibitor series was a derivative of benzimidazol-2-amine (compound 11), with a well-defined binding mode in *Tb*PTR1. Two types of substitutions were analysed, i.e. substituents at the C7 carbon of benzimidazole scaffold (6 inhibitors, *C7* set) and at the C4 position (4 inhibitors, *C4* set), the two substituent sets interacting predominantly with different subpockets of the *Tb*PTR1 protein.

For evaluation of the inhibitor–receptor interaction energies, we used the recently developed $E_{EL,MTP}^{(10)}+E_{Das}$ approximate model, including only multipole electrostatic and dispersive energy terms [7]. Combining these two terms is reasonable as they both are major long-range contributions to the intermolecular interaction energy. It is noteworthy that, despite the approximate approach, the model does not contain empirical parameters, i.e. it is parametrized only based on the theoretical SAPT results. The model, however, also involves the following approximations: (*i*) a reduced binding pocket representation that includes only the residues directly interacting with the inhibitors, and (*ii*) inhibitor–receptor interaction energy terms calculated in a pairwise manner, summing over contributions of each binding pocket residue. This calculation scheme, despite its simplicity, resulted in a good correlation with experimental inhibitory activities for both the *C7* and the *C4* sets of the *Tb*PTR1 inhibitors. Furthermore, this computationally efficient model was more accurate in predicting inhibitory activities of *Tb*PTR1 inhibitors than the extra-precision (XP) docking score of Glide (Schrödinger, Inc.). We also found that the replacement of the $E_{Das}$ function by the D3 correction to dispersionless—DFT could also be considered, since this approximation reflects the correlation energy contribution as accurately as the $E_{Das}$ energy. Unlike the latter, however, the D3 correction might be dependent on the choice of the functional and further validation is required to assess its range of applicability. Considering both the *C7* and *C4* sets of inhibitors, we obtained correlations with the experimental data for the $E_{EL,MTP}^{(10)}+E_{Das}$ model. For the more closed, confined *C4* subpocket, the $E_{EL,MTP}^{(10)}+E_{Das}$ model performed less well but gave results comparable to those obtained for FAAH inhibitors [7]: $R=-0.59$ versus $-0.67$, respectively. The lower correlation for the *C*4 inhibitor set may be due to the low number of compounds with a narrow range of experimental $p\text {K}_{\text {i}}^{app}$ values. We found that $E_{EL,MTP}^{(10)}+E_{Das}$ is better able to overcome deficiencies of the model of the ligand–receptor complex for the halogenated compound (inhibitor 24) than the interaction energy at the MP2 level of theory. However, the applicability of the $E_{EL,MTP}^{(10)}+E_{Das}$ model is limited by the omission of the $E_{EX}^{(10)}$ term, as seen for inhibitor 25 whose fragment is slightly too bulky to fit well in the *C4* subpocket. Finally, we have shown that the $E_{EL,MTP}^{(10)}+E_{Das}$ model for the *C7* subpocket could be transferred to the *C4* subpocket with refitting of the $\beta$ constant term. The model could therefore be applied to the prediction of novel compounds capable of reversible binding to the target enzyme. The observed partial transferability and favorable computational scaling of the $E_{EL,MTP}^{(10)}+E_{Das}$ model opens possibilities for future applications in lead optimization. The most accurate inhibitory activity predictions can be expected for a set of compounds with similar solvation energy and the binding poses that share a common characteristics. The transferability of the $E_{EL,MTP}^{(10)}+E_{Das}$ model between different subpockets should be investigated further for larger sets of compounds with more diverse chemical properties.

## Electronic supplementary material

Below is the link to the electronic supplementary material.

Supplementary material 1 (pdf 2091 KB)
