# Supplementary material for: Application of a simple quantum chemical approach to ligand fragment scoring for Trypanosoma brucei pteridine reductase 1 inhibition
Source: J Comput Aided Mol Des. 2017 Jul 7;31(8):715–28. doi: 10.1007/s10822-017-0035-4 (PMC5570812; doi:10.1007/s10822-017-0035-4)
Supplement: Supplementary file 1 — Supplementary material 1 (pdf 2091 KB) [file 10822_2017_35_MOESM1_ESM.pdf]

Supplementary Material

Application of a simple quantum chemical approach to ligand fragment scoring for Trypanosoma brucei pteridine reductase 1 inhibition

Wiktoria Jedwabny (equal first author),[†] Joanna Panecka (equal first author),[‡] Edyta Dyguda-Kazimierowicz,[*,†] Rebecca C. Wade,[‡,¶] and W. Andrzej Sokalski[†]

†*Department of Chemistry, Wrocław University of Science and Technology, Wrocław, Poland*

‡*Molecular and Cellular Modeling Group, Heidelberg Institute for Theoretical Studies (HITS), Heidelberg, Germany*

¶*Center for Molecular Biology (ZMBH), DKFZ-ZMBH Alliance, and Interdisciplinary Center for Scientific Computing (IWR), Heidelberg University, Heidelberg, Germany*

E-mail: Edyta.Dyguda@pwr.edu.pl

Estimation of the K_i^{app} value of the pure compound 28

The apparent inhibition constant K_i^{app} of the *impure* compound 28, i.e. of a mixture of 75% of compound 28 and 25% of compound 33 was reported by Spinks et al.[1] The K_i^{app} determined is 0.46 μM for the 28/33 mixture,[1] and 0.65 μM for compound 33 alone.[2] The K_i^{app} of pure compound 28 is unknown, but it should be lower than that of compound 33, because K_i^{app} for the 28/33 mixture is lower than for compound 33 alone. Furthermore, since compound 28 is a stronger inhibitor than compound 33, and a major component of the measured mixture (75%), its K_i^{app} value should not dramatically change with respect to the value estimated for the mixture. Nevertheless, we here estimate the K_i^{app} value for compound 28.

Assuming methodological consistency between ref.[1] with ref.[2] (the method of K_i^{app} determination was not clearly stated in ref.[1]), the inhibitors were considered as tight binding, and the associated theory was applied for K_i^{app} determination.[3,4] Williams and Morrison[3] state that: *A reversible tight-binding inhibitor is one that exerts its reversible inhibitory effect on an enzyme-catalyzed reaction at a concentration comparable to that of the enzyme.* However, the inhibitory activities of compounds 28 and 33 are in the micromolar range, whereas the concentration of the TbPTR1 enzyme in the experiments referenced by Spinks et al.[5] is 4.8 nM, so we assume that the inhibitors should be considered as classical, rather than tight-binding, inhibitors. Therefore, to estimate the inhibitory activities, we may use the equilibrium equation for a reversible linear inhibitor in a competitive assay for an enzyme characterized by Michaelis–Menten kinetics. Following Ref. 6, for a single inhibitor I, substrate S, Michaelis–Menten constant K_m and maximal reaction rate V_{max}, the enzymatic reaction rate v is:

$$v([I]) = \frac{V_{max}[S]}{[S] + K_m(1 + \frac{[I]}{K_i})}, \tag{1}$$

where K_i is the inhibition constant of inhibitor I.

As derived by Yonetani and Theorell[7], the equilibrium equation for two reversible linear inhibitors I_1 (compound 33) and I_2 (compound 28) in a competitive assay is:

$$v_{1,2}([I_1],[I_2]) = \frac{V_{max}[S]}{[S] + K_m(1 + \frac{[I_1]}{K_{i1}} + \frac{[I_2]}{K_{i2}} + \frac{[I_1][I_2]}{\alpha_{1,2}K_{i1}K_{i2}})} \tag{2}$$

$\alpha_{1,2}$=1 if the inhibitor effects are independent, $\alpha_{1,2}$>1 if the inhibitors hinder each other, and $\alpha_{1,2} = \infty$ when they are ideally mutually exclusive (ideally competitive). We assume that the inhibitors are ideally competitive, then $\alpha_{1,2} = \infty$, and

$$v_{1,2}([I_1],[I_2]) = \frac{V_{max}[S]}{[S] + K_m(1 + \frac{[I_1]}{K_{i1}} + \frac{[I_2]}{K_{i2}})} \tag{3}$$

Assuming that v is measured in the experiment, we can combine Eq. 1 and 3, by stating that $v_{1,2}([I_1],[I_2]) = v([I_1]+[I_2])$:

$$\frac{V_{max}[S]}{[S] + K_m(1 + \frac{[I_1]}{K_{i1}} + \frac{[I_2]}{K_{i2}})} = \frac{V_{max}[S]}{[S] + K_m(1 + \frac{[I_1]+[I_2]}{K_{i(mix)}})}$$

$$\frac{[I_1]}{K_{i1}} + \frac{[I_2]}{K_{i2}} = \frac{([I_1]+[I_2])}{K_{i(mix)}} \tag{4}$$

We know that $[I_2] = 3[I_1]$ (assuming that $[I_1]$ is the concentration of compound 33 and 25% of this compound is in the mixture with compound 28, the concentration of which is $[I_2]$), so:

$$\frac{1}{K_{i1}} + \frac{3}{K_{i2}} = \frac{4}{K_{i(mix)}} \tag{5}$$

Following Spinks et al.[1]: $K_i^{app} = K_i(1 + \frac{[S]}{K_m})$

When $[S]$ and K_m are constants of the experiment, we can write:

$$K_i^{app} = \gamma K_i, \tag{6}$$

where γ=const.

Using Eq. 6, Eq. 5 becomes:

$$\frac{1}{K_{i1}^{app}} + \frac{3}{K_{i2}^{app}} = \frac{4}{K_{i(mix)}^{app}} \Rightarrow K_{i2}^{app} = \frac{3}{\frac{4}{K_{i(mix)}^{app}} - \frac{1}{K_{i1}^{app}}} \tag{7}$$

$K_{i1}^{app}(c33) = 0.65\mu M$

$K_{i(mix)}^{app}(75\%c28 + 25\%c33) = 0.46\mu M$

$$K_{i2}^{app}(c28) = \frac{3}{\frac{4}{0.46} - \frac{1}{0.65}} = 0.42\mu M$$

Per-residue additivity of the $E^{(10)}_{EL,MTP} + E_{Das}$ model

Given that we have two systems:

- The inhibitor set *C7* interacts significantly with residues $R_1, \ldots, R_l, \ldots, R_k$ $(1 \leq l \leq k)$ in terms of $E^{(10)}_{EL,MTP} + E_{Das}$ energy. The interactions with other residues are similar for all the *C7* inhibitors and thus omitted from the system.

- Another inhibitor set *C4* interacts significantly in terms of $E^{(10)}_{EL,MTP} + E_{Das}$ energy with other residues $R_l, \ldots, R_k, \ldots, R_m$ $(l \leq k \leq m)$. Other residues are omitted as above.

We may construct a common linear model for the two sets of inhibitors, *C4* and *C7*. We need to include both sets of residues in the binding pocket system, i.e. $R_1, \ldots, R_m$. For the inhibitor $INH_n \in$(*C4*,*C7*):

$$pK_i^n = \alpha \sum_{j=1}^{m} (E_{Das} + E^{(10)}_{EL,MTP})^n_{R_j} + \beta, \tag{8}$$

where $(E^{(10)}_{EL,MTP} + E_{Das})^n_{R_j}$ is the interaction energy calculated for inhibitor INH_n and residue R_j, and pK_i^n is inhibitory activity predicted for INH_n.

Assuming that all inhibitors from the *C7* set interact weakly or similarly with $R_{k+1}, \ldots, R_m$ (these interactions should not affect the activity differences in the *C7* set; this assumption certainly holds for all short-range terms like exchange repulsion, but it may be less valid for long-range electrostatic multipole interactions involving charged amino acid residues), then for each inhibitor $INH_{n1} \in$*C7*: $\alpha \sum_{j=k+1}^{m} (E_{Das} + E^{(10)}_{EL,MTP})^{n1}_{R_j} + \beta = const_1$, and:

$$pK_i^{n1} = \alpha \sum_{j=1}^{k} (E_{Das} + E^{(10)}_{EL,MTP})^{n1}_{R_j} + \beta + const_1, \tag{9}$$

All inhibitors from the *C4* set interact weakly or similarly with $R_1, \ldots, R_{l-1}$. Therefore, for each inhibitor $INH_{n2} \in C4$: $\alpha \sum_{j=1}^{l-1}(E_{Das} + E_{EL,MTP}^{(10)})_{R_j}^{n2} + \beta = const_2$, and:

$$pK_i^{n2} = \alpha \sum_{j=l}^{m}(E_{Das} + E_{EL,MTP}^{(10)})_{R_j}^{n2} + \beta + const_2, \tag{10}$$

Note that the two linear models for the two sets of inhibitors, *C4* and *C7* have the same gradient α. In fact, the values of the gradient α, as well as the constant β, can be expected to differ between the two models due to inaccuracies and inconsistencies in the modelling procedure and the experimental data.

Interaction energy decomposition according to Hybrid Variation–Perturbation Theory

Following the Hybrid Variation–Perturbation Theory (HVPT)[8,9] interaction energy calculated at the Møller–Plesset second–order level of theory, E_{MP2}, can be partitioned into the following contributions:

$$E_{MP2} = E^{(10)}_{EL,MTP} + E^{(10)}_{EL,PEN} + E^{(10)}_{EX} + E^{(R0)}_{DEL} + E^{(2)}_{CORR} \tag{11}$$

$E^{(10)}_{EL,MTP}$ refers to the electrostatic multipole component estimated from an atomic multipole expansion:[10]

$$\Delta E^{k \leqslant L}_{EL,MTP} = \sum_{k_a} \sum_{k_b} \boldsymbol{M}^{(k_a)}_{A}[k_a]\boldsymbol{T}^{(k_a+k_b)}[k_b]\boldsymbol{M}^{(k_b)}_{B} \tag{12}$$

where $k_a + k_b \leqslant L$, $\boldsymbol{M}$ are rank k atomic multipoles for interacting molecules or molecular fragments A and B, and $\boldsymbol{T}$ is the Cartesian interaction tensor containing the partial derivatives of $|\boldsymbol{R}_{ab}|^{(-1)}$ of rank $k_a + k_b$, where $\boldsymbol{R}_{ab}$ is the interatomic vector between atoms a and b in A and B molecules, respectively. $E^{(10)}_{EL,MTP}$ is obtained from atomic multipole moments[11] derived from RHF monomer wavefunctions.

$E^{(10)}_{EL,PEN}$ from Eq. 11 is the electrostatic penetration energy, calculated from the following expression: $E^{(10)}_{EL,PEN} = E^{(10)}_{EL} - E^{(10)}_{EL,MTP}$, with the first–order electrostatic energy, $E^{(10)}_{EL}$, calculated according to the formula given in Eq. 13:

$$E^{(10)}_{EL} = \frac{< \psi_A\psi_B|\hat{H}_{AB} - \hat{H}_A - \hat{H}_B|\psi_A\psi_B >}{< \psi_A\psi_B|\psi_A\psi_B >} \tag{13}$$

where A and B denote the unbound molecules or molecular fragments, AB represents their dimeric complex, and $\hat{H}_X$ and ψ_X stand for the Hamiltonian and the wave function of X ($X = A$, B, or AB), respectively.

Subsequently, the first–order exchange energy $E_{EX}^{(10)}$ term in Eq. 11 is calculated from the first–order Heitler–London energy, $E^{(10)}$: $E_{EX}^{(10)} = E^{(10)} - E_{EL}^{(10)}$, with $E^{(10)}$ defined by the formula:

$$\begin{aligned} E^{(10)} &= \frac{< \hat{A}\psi_A\psi_B|\hat{H}_{AB}|\hat{A}\psi_A\psi_B >}{< \hat{A}\psi_A\psi_B|\hat{A}\psi_A\psi_B >} \\ &- \frac{< \psi_A|\hat{H}_A|\psi_A >}{< \psi_A|\psi_A >} - \frac{< \psi_B|\hat{H}_B|\psi_B >}{< \psi_B|\psi_B >} \end{aligned} \tag{14}$$

where $\hat{A}$ denotes the antisymmetry operator. The higher–order delocalization energy, $E_{DEL}^{(R0)}$, encompassing classical induction and charge transfer terms, is calculated as: $E_{DEL}^{(R0)} = E_{SCF} - E^{(10)}$, where E_{SCF} is the counterpoise–corrected self-consistent field variational energy. Finally, the correlation term, consisting mostly of the dispersion, exchange–dispersion and intra-molecular correlation contributions, is defined as: $E_{CORR}^{(2)} = E_{MP2} - E_{SCF}$.

In the above equations, the zero value of the second superscript denotes uncorrelated interaction energy contributions (i.e., zeroth–order electron fluctuation perturbation [12]), whereas the $E_{CORR}^{(2)}$ term represents the inter- and intra-molecular correlation contribution.

Supplementary Tables

Table S1: The total interaction energy[a] at the MP2 level of theory and corresponding $E^{(2)}_{CORR}$ energy obtained with the 6-311++G(2d,2p) and 6-311G(d) basis sets with diffuse functions on the s and p orbitals of the chlorine atom of fr-29.

Inhibitor	$p\mathrm{K}_i^{app}$ [b]	E_{MP2}		$E^{(2)}_{CORR}$	
		6-311++G(2d,2p)	6-311G(d)[c]	6-311++G(2d,2p)	6-311G(d)[c]
fr-32	8.2	-14.1	-9.5	-21.4	-16.5
fr-30	7.3	-13.4	-8.8	-17.9	-13.3
fr-31	7.0	-13.1	-7.9	-21.8	-16.8
fr-29	6.3	-6.6	-4.0	-11.2	-8.5
fr-33	6.2	-9.2	-6.4	-12.6	-9.7
fr-11	6.1	-6.3	-4.1	-10.1	-7.6
R[d]		-0.89	-0.89	-0.86	-0.86
N_{pred}[e]		86.7	86.7	66.7	66.7

[a]In units of $\mathrm{kcal \cdot mol^{-1}}$.
[b]Experimental affinity values are taken from Ref. 1.
[c]With diffuse functions on the s and p orbitals of the chlorine atom of fr-29.
[d]Pearson correlation coefficient between the calculated energy and the experimental inhibitory activity.
[e]Percentage of concordant pairs [%].

Table S2: Per-residue interaction energy[a] at the consecutive levels of theory: *C7* model.

Inhibitor	Residue	$E^{(10)}_{EL,MTP}$	$E^{(10)}_{EL}$	$E^{(10)}$	E_{SCF}	E_{MP2}	E_{Das}
fr-32	F171	-1.1	-2.0	2.1	1.5	-2.5	-5.1
	F97	-1.4	-3.1	4.2	3.3	-1.7	-6.3
	M213	-0.1	-0.4	1.2	1.0	-0.2	-1.8
	P210	0.1	-0.1	0.2	0.2	-0.6	-1.1
	W221–E217[b]	-1.9	-2.8	3.1	1.0	-4.4	-7.0
fr-30	F171	-1.2	-2.3	2.7	2.1	-2.7	-6.3
	F97	-1.3	-2.2	2.9	2.2	-1.9	-5.3
	M213	0.0	-0.2	1.1	1.0	0.1	-1.6
	P210	-0.3	0.0	0.0	0.0	-0.2	-0.3
	W221–E217[b]	-0.6	-2.2	0.9	-0.7	-4.1	-5.0
fr-31	F171	-0.5	-2.5	4.0	3.2	-2.8	-7.6
	F97	-1.2	-2.6	3.2	2.4	-2.0	-5.6
	M213	0.3	0.2	0.2	0.2	-0.1	-0.3
	P210	0.0	-0.1	-0.1	-0.1	-0.2	-0.2
	W221–E217[b]	1.3	-2.0	4.8	3.2	-2.8	-6.4
fr-29	F171	-1.3	-2.6	2.3	1.6	-2.6	-5.6
	F97	-1.1	-1.9	2.5	1.9	-1.7	-4.7
	M213	-0.1	-0.1	-0.1	-0.1	-0.2	-0.1
	P210	-0.1	-0.1	-0.1	-0.1	-0.1	-0.1
	W221–E217[b]	1.2	1.7	1.7	1.3	0.6	-0.5
fr-33	F171	-0.9	-2.4	3.2	2.4	-2.2	-6.1
	F97	-1.3	-2.5	3.0	2.2	-1.8	-5.2
	M213	0.2	0.2	0.2	0.1	0.0	-0.2
	P210	-0.2	-0.1	-0.1	-0.1	-0.1	-0.1
	W221–E217[b]	-0.7	-1.1	-1.0	-1.5	-2.3	-0.9
fr-11	F171	-1.2	-2.3	2.7	2.1	-2.1	-5.5
	F97	-1.1	-2.2	2.6	1.9	-1.4	-4.2
	M213	0.1	0.0	0.0	0.0	0.0	0.0
	P210	0.0	0.0	0.0	0.0	-0.1	0.0
	W221–E217	-0.3	-0.2	-0.2	-0.4	-0.5	-0.2

[a]In units of $\text{kcal} \cdot \text{mol}^{-1}$.
[b]Two residues treated in calculations as a dimer.

Table S3: Exchange and delocalization contributions to the interaction energy[a] of the *C7* set of inhibitor fragments.

Inhibitor	$p\mathrm{K}_\mathrm{i}^{app\,\mathrm{b}}$	$E_{EX}^{(10)}$	$E_{DEL}^{(R0)}$
fr-32	8.2	19.1	-3.8
fr-30	7.3	14.6	-3.1
fr-31	7.0	19.2	-3.3
fr-29	6.3	9.3	-1.7
fr-33	6.2	11.2	-2.0
fr-11	6.1	9.7	-1.5
R[c]		0.85	-0.94
N_{pred}[d]		26.7	73.3

[a]In units of $\mathrm{kcal} \cdot \mathrm{mol}^{-1}$.
[b]Experimental $p\mathrm{K}_\mathrm{i}^{app}$ values are taken from Ref. 1.
[c]Pearson correlation coefficient between the calculated energy and the experimental inhibitory activity.
[d]Percentage of concordant pairs [%].

Table S4: Inhibitory activities predicted with the best fitting equations obtained from Glide XP scoring and $E_{EL,MTP}^{(10)} + E_{Das}$ function.

Inhibitor	$p\mathrm{K}_\mathrm{i}^{app\,\mathrm{a}}$	$p\mathrm{K}_\mathrm{i}$ (Glide)	$p\mathrm{K}_\mathrm{i}$ ($E_{EL,MTP}^{(10)} + E_{Das}$)
fr-32	8.2	8.9	8.1
fr-30	7.3	6.5	7.5
fr-31	7.0	6.4	7.2
fr-29	6.3	6.7	5.9
fr-33	6.2	6.4	6.5
fr-11	6.1	6.2	6.0

[a]Experimental $p\mathrm{K}_\mathrm{i}^{app}$ values are taken from Ref. 1

Table S5: The total interaction energy[a] of the *C4* set of inhibitors at the consecutive levels of theory. In addition, energies for the $E^{(10)}_{EL,MTP} + E_{Das}$ and $E^{(10)}_{EL,MTP} + E^{(2)}_{CORR}$ models are given for the system with unprotonated Asp161.

Inhibitor	$p\mathrm{K}_\mathrm{i}^{app\,\mathrm{b}}$	$E^{(10)}_{EL,MTP}$	$E^{(10)}_{EL}$	$E^{(10)}$	E_{SCF}	E_{MP2}	$E^{(10)}_{EL,MTP} + E_{Das}$	$E^{(10)}_{EL,MTP} + E^{(2)}_{CORR}$
fr-25	6.5	-2.8	-16.5	32.6	20.5	-8.5	-48.7	-31.8
fr-28	6.4	4.4	-7.0	26.1	18.3	-4.1	-28.4	-18.1
fr-11	6.1	-3.1	-13.2	15.4	8.3	-8.2	-26.1	-19.6
fr-24	5.6	-1.8	-10.4	17.8	10.8	-8.7	-30.2	-21.2
R^c		0.29	-0.23	0.77	0.76	0.44	-0.49	-0.40
$N_{pred}{}^\mathrm{d}$		50.0	66.7	16.7	16.7	33.3	66.7	50.0

[a] In units of $\mathrm{kcal} \cdot \mathrm{mol}^{-1}$.
[b] Experimental affinity values are taken from Ref. 1.
[c] Pearson correlation coefficient between the calculated energy and the experimental inhibitory activity.
[d] Percentage of concordant pairs [%].

Table S6: Per-residue interaction energy[a] at the consecutive levels of theory: *C4* model. The data for the unminimized complex with compound 24 are marked by *.

Inhibitor	Residue	$E^{(10)}_{EL,MTP}$	$E^{(10)}_{EL}$	$E^{(10)}$	E_{SCF}	E_{MP2}	E_{Das}
fr-25	F171	-1.6	-2.8	3.7	2.9	-2.3	-7.3
	F97	-1.2	-2.3	2.7	2.0	-1.7	-4.6
	M163–V164[b]	0.1	-1.2	2.4	1.7	-0.6	-3.8
	P167	-0.6	-0.7	2.5	2.1	-0.1	-3.8
	Y174–N175[b]	0.1	-3.5	9.1	7.1	0.5	-12.0
	C168	0.4	-6.0	9.7	6.8	-0.7	-12.6
	D161	0.0	0.1	2.5	-2.1	-3.5	-1.8
	DH161[c]	1.1	-0.3	2.8	2.0	0.6	-3.0
fr-28	F171	-1.6	-2.7	3.5	2.7	-2.4	-7.3
	F97	-1.0	-2.1	2.6	1.9	-1.5	-4.3
	M163–V164[b]	-0.1	-0.1	-0.1	-0.1	-0.8	-0.8
	P167	0.3	0.2	0.5	0.4	-0.4	-1.2
	Y174–N175[b]	1.7	-0.6	5.7	4.6	-0.3	-7.9
	C168	1.4	-5.1	9.8	7.4	0.9	-10.3
	D161	3.6	3.6	4.1	1.5	0.3	-0.9
	DH161[c]	-0.9	-1.1	-0.5	-0.8	-1.5	-1.4
fr-11	F171	-1.2	-2.3	2.7	2.1	-2.1	-5.5
	F97	-1.1	-2.2	2.6	1.9	-1.4	-4.2
	M163–V164[b]	0.2	0.3	0.3	0.3	-0.2	-0.5
	P167	-0.2	-0.1	-0.1	-0.1	-0.2	-0.2
	Y174–N175[b]	1.3	0.1	2.7	2.4	-0.2	-3.1
	C168	0.2	-6.0	9.3	6.8	1.1	-8.7
	D161	-2.5	-3.0	-2.1	-5.0	-5.2	-0.8
	DH161[c]	1.6	0.7	2.0	1.4	0.5	-1.5
fr-24	F171	-0.8	-2.0	2.7	2.0	-2.5	-6.0
	F97	-0.7	-1.9	2.7	2.1	-1.3	-4.3
	M163–V164[b]	0.9	0.8	0.9	0.8	0.0	-0.8
	P167	-0.7	-0.5	-0.5	-0.5	-0.7	-0.6
	Y174–N175[b]	-1.8	-2.4	3.5	2.6	-1.4	-6.8
	C168	1.0	-4.9	7.2	5.3	-0.5	-8.9
	D161	0.3	0.5	1.3	-1.5	-2.3	-1.1
	DH161[c]	1.9	0.4	2.2	1.5	0.4	-2.2
fr-24*	F171	-0.9	-3.2	3.9	2.8	-2.3	-7.0
	F97	-1.2	-2.2	2.9	2.2	-1.4	-4.6
	M163–V164[b]	0.7	0.6	0.6	0.6	0.0	-0.6
	P167	-0.6	-0.5	-0.4	-0.5	-0.6	-0.5
	Y174–N175[b]	-1.5	-2.7	3.4	2.5	-0.2	-4.7
	C168	-0.4	-10.5	15.4	11.2	2.6	-15.3
	D161	-0.7	-0.3	0.0	-2.3	-2.7	-0.7
	DH161[b]	1.8	0.9	1.3	1.0	0.3	-1.2

[a]In units of $\text{kcal} \cdot \text{mol}^{-1}$.
[b]Two residues treated in calculations as a dimer.
[c]Protonated state of Asp161.

Table S7: Exchange, delocalization and correlation contributions to the interaction energy, and the E_{Das} energy^{a} of the *C4* set of inhibitor fragments, for the system with protonated Asp161. The data for the unminimized complex with compound 24 are marked by *.

Inhibitor	$p\mathrm{K}_{\mathrm{i}}^{app\,\mathrm{b}}$	$E_{EX}^{(10)}$	$E_{DEL}^{(R0)}$	$E_{CORR}^{(2)}$	E_{Das}
fr-25	6.5	49.8	-8.3	-29.0	-47.1
fr-28	6.4	33.2	-5.4	-21.8	-33.3
fr-11	6.1	29.0	-4.7	-17.2	-23.7
fr-24	5.6	29.2	-4.9	-19.9	-29.6
R^{c}		0.69	-0.67	-0.62	-0.61
$N_{pred}{}^{\mathrm{d}}$		16.7	83.3	83.3	83.3
fr-24*	5.6	44.7	-7.2	-21.6	-34.0
$\mathrm{R}^{*\mathrm{c}}$		-0.03	-0.05	-0.48	-0.42
$N_{pred}^{*}{}^{\mathrm{d}}$		33.3	66.7	83.3	66.7

^{a}In units of $\mathrm{kcal} \cdot \mathrm{mol}^{-1}$.
^{b}Experimental affinity values are taken from Ref. 1.
^{c}Pearson correlation coefficient between the calculated energy and the experimental inhibitory activity.
^{d}Percentage of concordant pairs [%].

Table S8: Pearson correlation coefficients (R) between E_{MP2} and $E_{EL,MTP}^{(10)} + E_{Das}$ for the *C4*–set inhibitors with exclusion of one inhibitor from the set. Positive R values denote correlation and negative values denote anti-correlation.

Excluded inhibitor	R
none (full *C4* set)	0.27
without fr-25	0.92
without fr-28	0.32
without fr-11	-1.00
without fr-24	0.49

Table S9: Comparison of the interaction energy models for the *C4* and *C7* inhibitor sets (*C4* set does not include inhibitor 25). Parameters are given for the linear model[a]: $E = \alpha\, p\mathrm{K}_\mathrm{i}^{app} + \beta$, where E represents a particular interaction energy model; β_2 is a parameter for the *C4* set, calculated assuming that α is the same as for the *C7* model (see text for details); * denotes the *C4* set with unminimized inhibitor 24. The plots for the selected models are shown in Fig. 8 in the main text.

	E_{MP2}	$E^{(10)}_{EL,MTP} + E_{Das}$
C7 set		
α	-2.6	-6.5
β	10.7	26.4
C4 set		
α	-2.6	-6.5
β_2	10.7	10.5
*C4** set		
α	-2.6	-6.5
β_2	12.1	8.4

[a] E and α, β, β_2 parameters are in units of $\mathrm{kcal \cdot mol^{-1}}$.

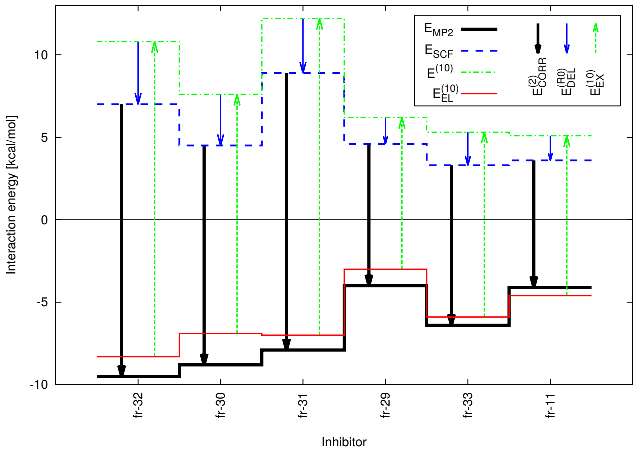

Figure S1: The components of the interaction energy. For each inhibitor fragment from the *C7* set, the total binding energy is given at the consecutive levels of theory represented by horizontal lines. The vertical arrows represent correlation, delocalization and exchange contributions to the MP2 binding energy and demonstrate mutual compensation of these interaction energy terms.

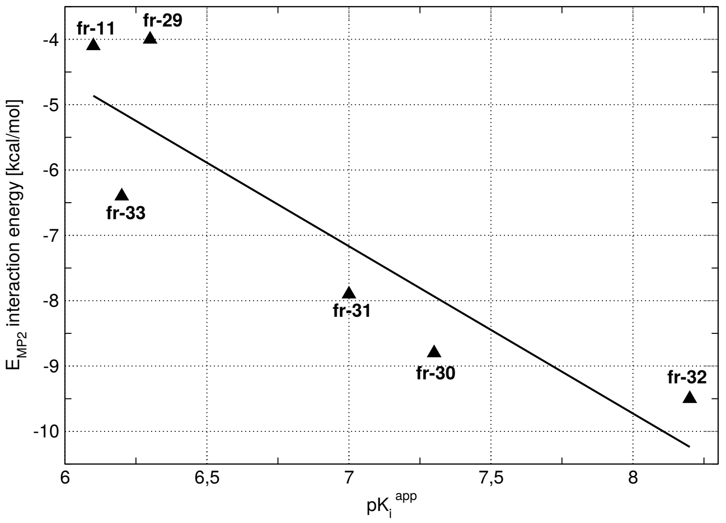

Figure S2: E_{MP2} interaction energy of the *C7* set of inhibitor fragments as a function of inhibitory activity.[1]

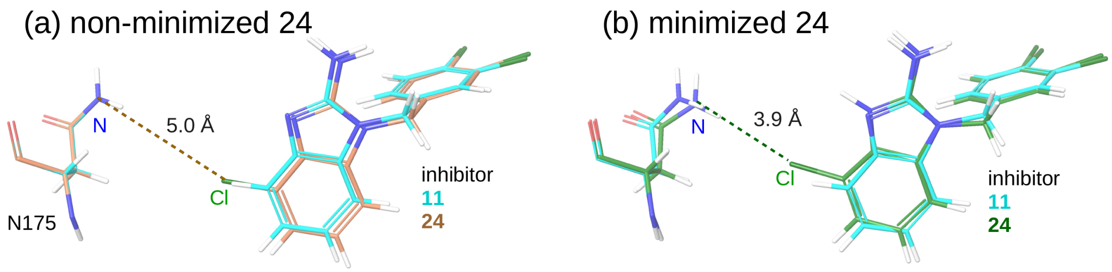

Figure S3: Comparison of the position of unminimized (a) and minimized (b) inhibitor 24 with respect to Asn175 residue. The pose of compound 11 with Asn175 from the modelled complex is shown as a reference.

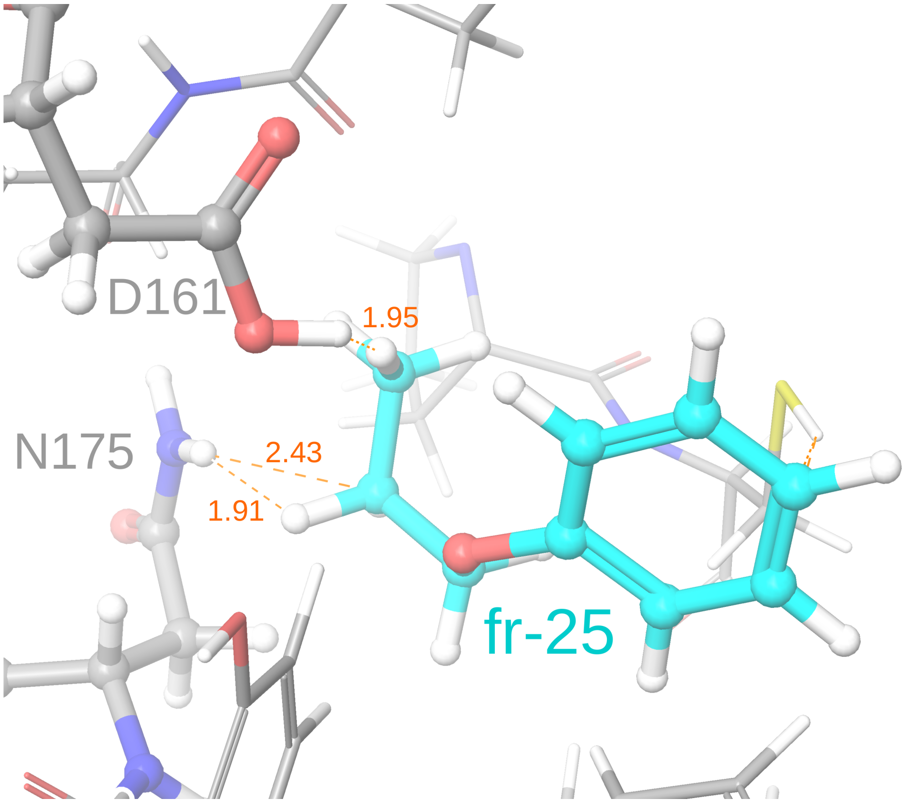

Figure S4: The docked pose of inhibitor 25 showing clashes of the substituent with the receptor (in orange, distances in Å).

References

(1) Spinks, D.; Ong, H. B.; Mpamhanga, C. P.; Shanks, E. J.; Robinson, D. A.; Collie, I. T.; Read, K. D.; Frearson, J. A.; Wyatt, P. G.; Brenk, R.; Fairlamb, A. H.; Gilbert, I. H. Design, Synthesis and Biological Evaluation of Novel Inhibitors of Trypanosoma brucei Pteridine Reductase 1. *ChemMedChem* **2011**, *6*, 302–308.

(2) Mpamhanga, C. P.; Spinks, D.; Tulloch, L. B.; Shanks, E. J.; Robinson, D. A.; Collie, I. T.; Fairlamb, A. H.; Wyatt, P. G.; Frearson, J. A.; Hunter, W. N.; Gilbert, I. H.; Brenk, R. One Scaffold, Three Binding Modes: Novel and Selective Pteridine Reductase 1 Inhibitors Derived from Fragment Hits Discovered by Virtual Screening. *J. Med. Chem.* **2009**, *52*, 4454–4465.

(3) Williams, J. W.; Morrison, J. F. The kinetics of reversible tight-binding inhibition. *Methods Enzymol.* **1979**, *63*, 437–467.

(4) Kuzmic, P.; Sideris, S.; Cregar, L. M.; Elrod, K. C.; Rice, K. D.; Janc, J. W. High-throughput screening of enzyme inhibitors: automatic determination of tight-binding inhibition constants. *Anal. Biochem.* **2000**, *281*, 62–67.

(5) Shanks, E. J.; Ong, H. B.; Robinson, D. A.; Thompson, S.; Sienkiewicz, N.; Fairlamb, A. H.; Frearson, J. A. Development and validation of a cytochrome c-coupled assay for pteridine reductase 1 and dihydrofolate reductase. *Anal. Biochem.* **2010**, *396*, 194–203.

(6) Dixon, M. The determination of enzyme inhibitor constants. *Biochem. J.* **1953**, *55*, 170–171.

(7) Yonetani, T.; Theorell, H. Studies on Liver Alcohol Hydrogenase Complexes. 3. Multiple Inhibition Kinetics in the Presence of Two Competitive Inhibitors. *Arch. Biochem. Biophys.* **1964**, *106*, 243–251.

(8) Sokalski, W. A.; Roszak, S.; Pecul, K. An efficient procedure for decomposition of the SCF interaction energy into components with reduced basis set dependence. *Chem. Phys. Lett.* **1988**, *153*, 153–159.

(9) Góra, R.; Sokalski, W. A.; Leszczyński, J.; Pett, V. The nature of interactions in the ionic crystal of 3-pentenenitrile, 2-nitro-5-oxo, ion(-1) sodium. *J. Phys. Chem. B* **2005**, *109*, 2027–2033.

(10) Sokalski, W. A.; Sawaryn, A. Correlated molecular and cumulative atomic multipole moments. *J. Chem. Phys.* **1987**, *87*, 526–534.

(11) Sokalski, W. A.; Poirier, R. A. Cumulative atomic multipole representation of the molecular charge distribution and its basis set dependence. *Chem. Phys. Lett.* **1983**, *98*, 86–92.

(12) Jeziorski, B.; Moszyński, R.; Szalewicz, K. Perturbation Theory Approach to Intermolecular Potential Energy Surfaces of van der Waals Complexes. *Chem. Rev.* **1994**, *94*, 1887–1930.
